# Dr. John F. Sanford

**Published:** 1869-04

**Authors:** 


DR. JOHN F. SANFORD.—In the last issue of our JOURNAL, we noticed the fact that Dr. J. F. Sanford, of this city, had abandoned his infirmary, and was about to quit the profession, having engaged in *Life Insurance and Patent Rights.* Having fully consummated his plans, he has entered his new field of labor, and may hereafter be found at No. 254 Broadway, New York. The Doctor's parting address to his friends calls loudly for a notice at our hands; but we have not time nor space in this issue to do the production justice. Should our feelings run in the proper channel ere another number of the JOURNAL appears, we will take pleasure in ventilating the Doctor and his new calling in a becoming manner; but for the present we must allow his name and invention to go unhonored and unsung, and can only wish him domestic happiness and filial comforts, and when his *sands* of life are about run out, may he find more *sand*.

